# An Enhanced Knowledge Salp Swarm Algorithm for Solving the Numerical Optimization and Seed Classification Tasks

**DOI:** 10.3390/biomimetics10090638

**Published:** 2025-09-22

**Authors:** Qian Li, Yiwei Zhou

**Affiliations:** 1 Guangdong Provincial Key Laboratory of Ornamental Plant Germplasm Innovation and Utilization, Environmental Horticulture Research Institute, Guangdong Academy of Agricultural Sciences, Guangzhou 510640, China; 2 Department of Computer Engineering, College of Engineering, Dongshin University, Naju 58245, Republic of Korea

**Keywords:** swarm intelligence, enhanced knowledge Salp Swarm Algorithm, Gaussian mutation strategy, dynamic mirror learning strategy, seed classification

## Abstract

The basic Salp Swarm Algorithm (SSA) offers advantages such as a simple structure and few parameters. However, it is prone to falling into local optima and remains inadequate for seed classification tasks that involve hyperparameter optimization of machine learning classifiers such as Support Vector Machines (SVMs). To overcome these limitations, an Enhanced Knowledge-based Salp Swarm Algorithm (EKSSA) is proposed. The EKSSA incorporates three key strategies: Adaptive adjustment mechanisms for parameters $c_{1}$ and α to better balance exploration and exploitation within the salp population; a Gaussian walk-based position update strategy after the initial update phase, enhancing the global search ability of individuals; and a dynamic mirror learning strategy that expands the search domain through solution mirroring, thereby strengthening local search capability. The proposed algorithm was evaluated on thirty-two CEC benchmark functions, where it demonstrated superior performance compared to eight state-of-the-art algorithms, including Randomized Particle Swarm Optimizer (RPSO), Grey Wolf Optimizer (GWO), Archimedes Optimization Algorithm (AOA), Hybrid Particle Swarm Butterfly Algorithm (HPSBA), Aquila Optimizer (AO), Honey Badger Algorithm (HBA), Salp Swarm Algorithm (SSA), and Sine–Cosine Quantum Salp Swarm Algorithm (SCQSSA). Furthermore, an EKSSA-SVM hybrid classifier was developed for seed classification, achieving higher classification accuracy.

## 1. Introduction

Swarm intelligence algorithms (SIAs) [1,2,3,4] are usually inspired by characteristics of the natural swarm of the animal, which can be applied to deal with complex optimization issues from modeling real-world problems. In addition, the behavior and characteristic of biological individuals are also considered in the design of the algorithm. Some widely SIAs include Particle Swarm Optimization (PSO) [1], Grey Wolf Optimizer (GWO) [5], Salp Swarm Algorithm (SSA) [6], Aquila Optimizer (AO) [7], Duck Swarm Algorithm (DSA) [3], etc. Expert in numerical optimization problems, engineering constrained optimization problems are also an effective method for verifying proposed algorithms. In addition, it can be abstracted as that all optimization problems can be solved by using SIAs and their variants in theory.

A Salp Swarm Algorithm (SSA) is a metaheuristic optimization technique modeled after the swarming and foraging behavior of salps in marine environments [6,8] The main steps are from the leader and followers, which has the advantages of simple structure and few parameters. As we know, the basic SSA has been researched with different strategies for optimization problems, which is used to avoid becoming stuck in local optima. Wang et al. [9] proposed a modified SSA to solve the node coverage task of wireless sensor networks (WSNs) with tent chaotic population initialization, T-distribution mutation, and adaptive position update strategy. Zhou et al. [10] designed an improved A* algorithm with SSA using a refined B-spline interpolation strategy, which is used for the path planning problem. Mahdieh et al. [11] designed an improved SSA via a robust search strategy and a novel local search method for solving feature selection (FS) tasks. Zhang et al. [12] proposed a cosine opposition-based learning (COBL) to modify SSA and used it to address the FS problem. Wang et al. [13] designed a spherical evolution algorithm with spherical and hypercube search by two-stage strategy. In addition, multi-perspective initialization, Newton interpolation inertia weight, and followers’ update model strategies were also used in the improved SSA. A Gaussian Mixture Model (GMM) via SSA [14] was proposed for data clustering of big data processing.Wang et al. [15] used symbiosis theory and the Gaussian distribution to improve SSA with better exploitation capabilities, which was applied to optimize multiple parameters in fuel cell optimization systems. However, the performance of the basic SSA should be improved by the knowledge-enhanced strategy, where novelty Gaussian mutation, dynamic adjustment of hyperparameter, and mirror learning strategies can be researched for specific tasks.

For the classification task, SIAs are usually utilized to optimize the hyperparameters of the classifier network [16,17,18]. An improved pelican optimization algorithm (IPOA) [16] was designed to optimize the combination model of variational mode decomposition (VMD) and long short-term memory (LSTM), which is used to forecast Ultra-Short-Term Wind Speed. Li et al. [17] proposed an improved Parrot Optimizer (IPO) with an aerial search strategy to train the multilayer perceptron (MLP), which enhanced the exploration and optimization ability of the basic pelican optimizer. Its performance was evaluated using the CEC benchmark function and the data set of the oral English teaching quality classification. Song et al. [18] proposed a modified pelican optimization algorithm with multi-strategies and used it for a high-dimensional feature selection task through K-nearest neighbor (KNN) and support vector machine (SVM) classifiers. Panneerselvam et al. [19] proposed a dynamic salp swarm algorithm and weighted extreme learning machine to deal with the imbalance task in the classification dataset with a higher accuracy. Wang et al. [20] proposed a hierarchical and distributed strategy to enhance the Gravitational Search Algorithm, which is inspired by the structure of Multi-Layer Perceptrons (MLPs), resulting in significantly improved performance compared to existing methods. Yang et al. [21] proposed a self-learning salp swarm algorithm (SLSSA) to train the MLP classifier using UCI datasets; its performance was also verified by CEC2014 benchmark functions with a longer computational time than basic SSA.

In the field of smart agriculture, the application of SIAs can effectively enhance the efficiency of plant disease diagnosis [22] and seed classification [23,24] by classifiers, significantly improve work efficiency, and thus reduce economic costs. Pranshu et al. [25] used the five most popular machine learning approaches to the Rice varieties classification problem. Din et al. [26] used a Deep Convolutional Neural Network to identify rice grain varieties via the pre-trained strategy, named RiceNet, which can have better prediction accuracy than traditional machine learning (ML) methods. Iqbal et al. [27] used three lightweight networks to perform the rice varieties classification task, which can extend to mobile devices. However, the deep neural network method needs more computing resources than the joint ML classifier and the SIA method.

To address the aforementioned challenges, this study proposes an enhanced-knowledge Salp Swarm Algorithm (EKSSA) to optimize the critical parameters of SVM in seed classification tasks. The proposed algorithm incorporates several strategic improvements to enhance its optimization performance. Specifically, adaptive adjustment mechanisms for parameters $c_{1}$ and α are introduced to effectively balance the exploration and exploitation capabilities of the salp population. Furthermore, a novel position update strategy based on Gaussian walk theory is implemented after the basic position update phase to significantly enhance the global search ability of individual salps. Additionally, a dynamic mirror learning strategy is designed to prevent premature convergence to local optima by creating mirrored search regions, thereby substantially improving local search efficiency. The effectiveness of the EKSSA is comprehensively evaluated through experiments on thirty-two CEC benchmark functions and two practical seed classification datasets, demonstrating its superior performance in both optimization accuracy and classification tasks. In summary, the contributions of the designed EKSSA are as follows:

- To enhance the performance of the basic SSA, an enhanced-knowledge Salp Swarm Algorithm (EKSSA) is proposed, and its effectiveness is rigorously evaluated through comparisons with other state-of-the-art optimization algorithms.
- Exploration and exploitation of the follower balances using different adjustment strategies for the parameters $c_{1}$ and α by the exponential function.
- A novel Gaussian mutation strategy and a dynamic mirror learning strategy are introduced to enhance global search capability and prevent EKSSA from becoming trapped in local optima.
- Many CEC benchmark functions are applied to evaluate the performance of the designed EKSSA, and two seed classification datasets are also utilized by the combination of EKSSA and the SVM algorithm which is named EKSSA-SVM.

The remainder of this paper is structured as follows: Section 2 presents the basic SSA. Section 3 introduces the mathematical model of the proposed EKSSA. Section 4 presents and discusses the experimental results of comparative algorithms. In Section 5, the application of EKSSA for optimizing hyperparameters of SVM in seed classification is described. Finally, Section 6 concludes the study and suggests potential future research directions.

## 2. The Basic Salp Swarm Algorithm

Salp Swarm Algorithm (SSA) is a metaheuristic optimization technique modeled after the swarming and foraging behavior of salps; the main steps are from the leader and followers. The mathematical model of the behavior corresponds to the optimization process of the proposed SSA. There are two key stages of the basic SSA: Population Initial Stage and Position Update Stage.

### 2.1. Population Initial Stage

The optimization problem is assumed to have a *D*-dimensional search space, and the initial positions of the salp population are defined as:

(1) $$X_{i,j}=rand_{i,j}\cdot \left(UB_{i,j}-LB_{i,j}\right)+LB_{i,j}$$

where $X_{i,j}$ denotes the initial position of the slap, i=1,2,…,NP, j=1,2,…,Dim. NP is the number of initial solutions and Dim is the dimension of the issue. $rand_{i,j}$ represents a random value in (0, 1) distributed uniformly. $UB_{i,j}$ and $LB_{i,j}$ indicate the upper and lower boundary values of the search space, respectively.

### 2.2. Position Update Stage

In the individual search process, the food source *F* for the slap is treated as the target, which is the optimal objective. In the search space, the position is updated by:

(2) $$X_{j}^{leader}=\left\{\begin{array}{cc} \; & F_{j}+c_{1}\left(\left(UB_{j}-LB_{j}\right)c_{2}+LB_{j}\right),if\;c_{3}>0.5\\ \; & F_{j}-c_{1}\left(\left(UB_{j}-LB_{j}\right)c_{2}+LB_{j}\right),if\;c_{3}\leq 0.5 \end{array}\right.$$

where $X_{j}^{leader}$ represents the position of the leader in the *j*th dimension. $F_{j}$ indicates the *j*th dimensional food source. $c_{1},c_{2}$ and $c_{3}$ denote a random value in (0, 1) according to the Gaussian law.

After the leader position with respect to the food source, the exploration and exploitation of the follower balances by the parameter $c_{1}$, which is defined as follows:

(3) $$c_{1}=2\cdot exp\left(-{\left(\frac{4\cdot l}{T_{max}}\right)}^{2}\right)$$

where *l* denotes the current iteration. $T_{max}$ indicates the maximum number. Notably, the follower’s position is updated by:

(4) $$X_{j}^{i}=\frac{1}{2}\left(X_{j}^{i}+X_{j}^{i-1}\right)$$

where $X_{j}^{i}$ denotes the position of the *i*th follower in the *j*th dimension when i≥2.

## 3. The Proposed Enhanced Knowledge Salp Swarm Algorithm

In this study, to overcome the shortage of SSA falling into local optimum, we use the improved method to optimize hyperparameter of the SVM classifier for seed classification tasks. We propose an adjustment strategy for the parameter α to balance the optimization process of the follower position. In addition, the Gaussian mutation strategy and the mirror learning strategy are employed to enhance the overall performance of the proposed EKSSA. Moreover, the position update strategy of the follower is also a novel approach. The following introduces in detail the process of the improved strategies.

**Figure 1:**
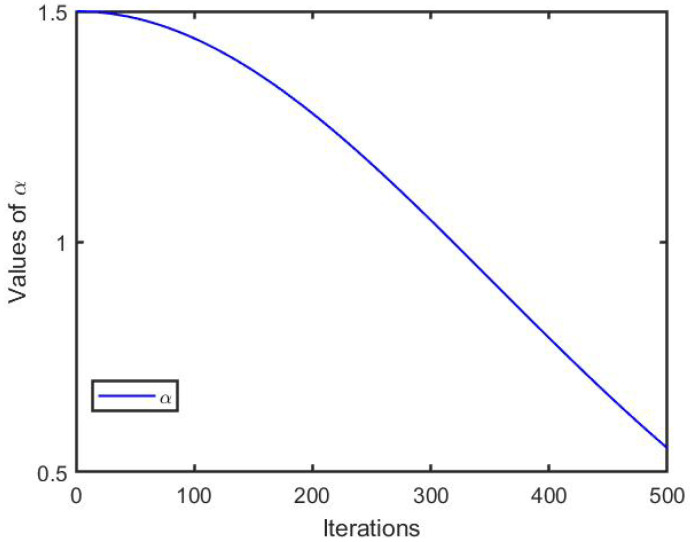
The curve of the parameter α.

To enhance the performance between exploration and exploitation of the follower, the adjustment strategy of the parameter α is calculated by:

(5) $$\alpha =1.5\cdot exp\left(-{\left(\frac{l}{T_{max}}\right)}^{2}\right)$$

where α denotes the adjustment strategy parameter, which is used to balance the follower position. *l* indicates the current iteration. $T_{max}$ is the maximum number. The hyperparameter α curve is depicted in Figure 1, because nonlinear strategies can effectively enhance the search ability of EKSSA during the optimization process. In the early stage of the slap search, a larger α value (α>1) can help followers obtain a better search space. In the later stage of slap search, reducing the α value (α<1) can help followers search for the optimal value of the optimization problem.

Then, the follower’s position from Equation (4) can be redefined as:

(6) $$X_{j}^{i}=\alpha \cdot \frac{1}{2}\left(X_{j}^{i}+X_{j}^{i-1}+r_{1}\cdot F_{j}\right)$$

where $X_{j}^{i}$ denotes the position of the *i*th follower in the *j*th dimension when the i≥2. $r_{1}$ is a random number in (0,1) according to the Gaussian law. $F_{j}$ indicates the *j*th dimensional food source, which is the best position during the search process of the individual.

Notably, a new Gaussian mutation strategy is proposed to avoid falling into local optimum, and its expression is:

(7) $$X_{j}^{i}=Gaussian\left(X_{j}^{i},\theta \right)\cdot \left|X_{j}^{i}-F_{j}\right|$$

where θ denotes the standard deviation of Gaussian variation and it is set to 0.5. $X_{j}^{i}$ denotes the position of the *i*th follower in the *j*th dimension. $F_{j}$ indicates the *j*th dimensional food source. The expression of the Gaussian walk $Gaussian(X_{j}^{i},\theta )$ is defined as:

(8) $$Gaussian\left(X_{j}^{i},\theta \right)=\frac{1}{\sqrt{2\pi \theta }}exp\left(-\frac{{\left(F_{j}-X_{j}^{i}\right)}^{2}}{2\theta^{2}}\right)$$

In addition, the mirror learning strategy is employed to prevent the EKSSA from converging on local optima, thereby strengthening its global search performance, which is defined as:

(9) $$X_{j}^{i}=r_{2}\cdot \frac{UB_{j}+LB_{j}}{2}+\frac{UB_{j}+LB_{j}}{2k}-\frac{X_{j}^{i}}{k}$$

where $r_{2}$ is a random number in (0,1) according to the Gaussian law. *k* denotes the scaling factor of the mirror learning strategy. $UB_{i,j}$ and $LB_{i,j}$ indicate the upper and lower boundary values of the individual, respectively. The adjust strategy of the *k* is defined as:

(10) $$k=\left\{\begin{array}{cc} \; & {\left(1+\sqrt{\frac{l}{T_{max}}}\right)}^{5},if\;r_{3}>0.5\\ \; & 1,if\;r_{3}\leq 0.5 \end{array}\right.$$

where $r_{3}$ denotes a random number in (0,1) according to the Gaussian law. *l* is the current iteration. $T_{max}$ is the maximum number.

### 3.1. Computational Complexity Analysis

The test platforms can influence the consumption of optimization time for the same algorithm, which means that the designed EKSSA should be analyzed. Assuming that *N* is the population size of the EKSSA, *T* indicates the maximum number of iterations, and *D* is the dimension. The computational complexity of the proposed EKSSA algorithm is analyzed as follows: the initialization of the salp population requires O(ND) operations; the position update in the basic global and local search phase has a complexity of O(NDlogD); the Gaussian mutation and mirror learning strategies contribute an additional 2O(ND) to the update complexity. Furthermore, the complexity of the fitness sorting is O(NlogN). Therefore, the overall computational complexity of the EKSSA can be expressed as:

(11) $$O\left(EKSSA\right)=O\left(ND\right)+O\left(T\right)\cdot \left(O\left(NDlogD\right)+2\cdot O\left(ND\right)+O(NlogN)\right)$$

However, the computational complexity of the basic SSA is:

(12) $$O\left(SSA\right)=O\left(ND\right)+O\left(T\right)\cdot \left(O\left(NDlogD\right)+O(NlogN)\right)$$

### 3.2. Flowchart and Pseudo-Code of the EKSSA

**Figure 2:**
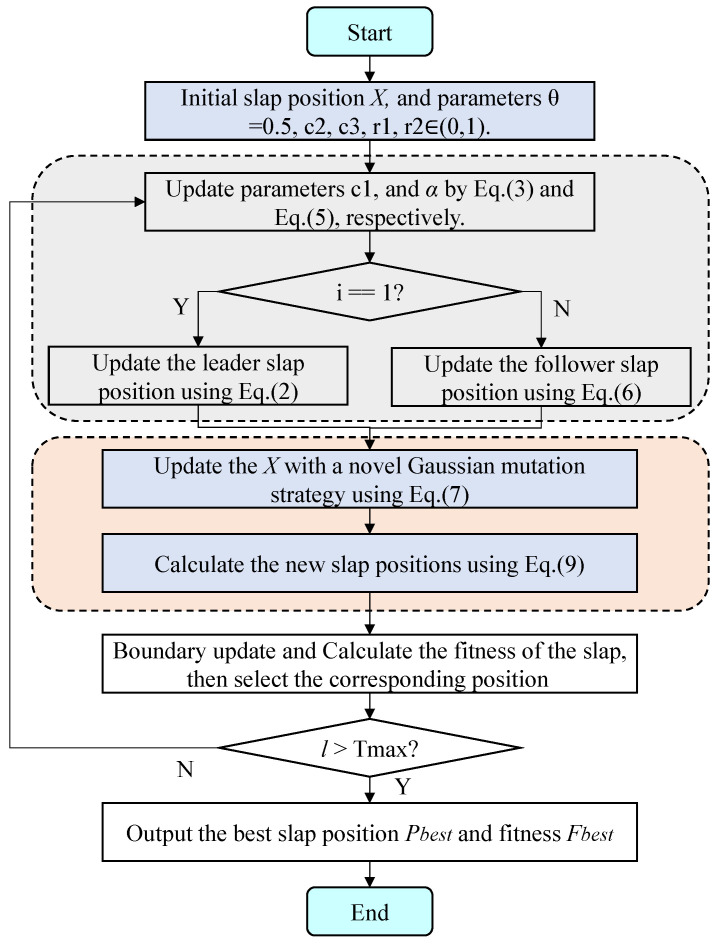
The flowchart of the designed EKSSA.

Figure 2 illustrates the flowchart of the EKSSA algorithm, detailing its optimization process. From Figure 2, it can be summarized in four stages. Stage 1 is the initialization of the slap position; Stage 2 includes parameter update, leader and follower position update of the proposed EKSSA; Stage 3 involves individual position update by the Gaussian mutation and mirror learning strategy. Stage 4 represents the best population of slaps corresponding to fitness during the optimization process. After the $T_{max}$ iterations of the designed method, the best solution and fitness value are generated.

To understand the main architecture of the method, the pseudo-code of the designed EKSSA is displayed in Algorithm 1. In particular, input, output, and the main code of the EKSSA are listed.
**Algorithm 1:** Pseudo-code of EKSSA.
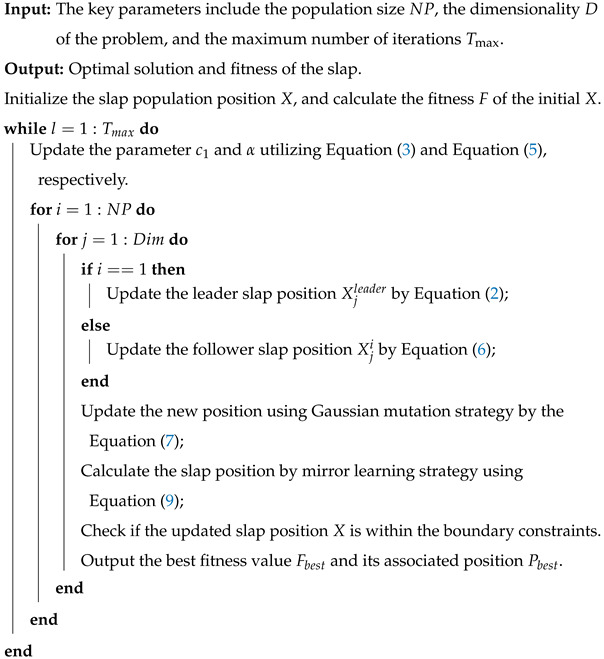


## 4. Results and Analysis

This section details the experimental setup, including the selected benchmark functions, algorithm hyperparameters, and the presentation of results from the CEC benchmarks and boxplot analyses.

### 4.1. Selected Benchmark Test Functions

A comprehensive evaluation of the proposed algorithm’s performance was conducted on 32 functions from the CEC benchmark suite [28,29,30,31], where F1 to F6 are unimodal (U), F7 to F10 are multimodal (M), and F11 to F18 are fixed-dimensional (M) functions, respectively. The dimension of F1 to F10 was wet at 30. Table 1 details the 18 functions. Table depicts eight functions (F19 to F26) from CEC2017 [30] and six functions (F27 to F32) from CEC2022 [31], respectively. For the experiment in this study, Matlab 2018a was the platform with a Windows 10 system, 16 GB memory, and Intel(R) (Santa Clara, CA, USA.) Core (TM) i5-10210U CPU @2.11 GHz.

### 4.2. Hyperparameter Settings

In our work, the performance and effectiveness of the designed EKSSA were assessed through a comprehensive suite of 32 numerical optimization benchmark functions. The comaprison algorithms are Randomized Particle Swarm Optimizer (RPSO) [32], Grey Wolf Optimizer (GWO) [5], Archimedes Optimization Algorithm (AOA) [33], Hybrid Particle Swarm Butterfly Algorithm (HPSBA) [34], Aquila Optimizer (AO) [7], Honey Badger Algorithm (HBA) [35], Salp Swarm Algorithm (SSA) [6], Sine–Cosine Quantum Salp Swarm Algorithm (SCQSSA) [36], and the proposed EKSSA. Notably, Table 2 presents the hyperparameter settings of the comparison approaches. For the optimization problem, the size of the population is set to 30 of all comparison approaches in this study, that is, NP=30. Each test function is executed independently 30 times, and the maximum number of iterations $T_{max}$ serves as the condition for optimization termination. $T_{max}$ is set to 1000 in this study.

### 4.3. Analysis of CEC Benchmark Function Results

From Table 3 containing the results of F1, F2, F3, F7, and F9, the proposed EKSSA can obtain the theoretical optimum, where Best, Worst, Mean, and Std are all the best. The smaller the STD value, the better the stability of the algorithm’s optimization of the comparison methods. For the fixed functions F16, F17, and F18, the EKSSA achieves the theoretical optimal value with the smallest Std value in the comparison methods. For the RPSO, its performance is better than that others on F13, F14, F15 under the best Std with theoretical optimum. In addition, the HPSBA can also obtain the same result as EKSSA in F1, F2, F3, F7, and F9; however, its performance should be improved on other benchmark functions. For F7 and F8, HBA, AO, HPSBA, SCQSSA and EKSSA have better results than others. For F9, HBA, AO, AOA, HPSBA, SCQSSA and EKSSA achieve the theoretical optimal value. From Table 3, the results show that EKSSA holds the top rank against the eight other methods over the suite of 18 test functions, and the overall ranking is EKSSA > HBA > AO > AOA > GWO > RPSO > SSA > HPSBA > SCQSSA. Also, Table 4 shows *p*-values of the comparison algorithms for F1 to F18 by the WSR test.

From Table 5, we report results on a collection of 14 test functions drawn from the CEC2017 and CEC2022 benchmarks with Mean, Std, Time, and *p*-value by the WSR test. For the Mean of F19, F20, F22, F23, F31, F32, the proposed EKSSA can obtain the best values comapred to other comparison algorithms. RPSO can obtain the best result from the Mean of F26, F27, and F29 compared to others. The performance of the EKSSA on complex numerical optimization problems is thus demonstrably effective. This is validated by the Friedman test results presented in Table 6, which ranks the algorithms based on their performance in 14 test functions of the CEC2017 and CEC2022 benchmark suites. The overall ranking is EKSSA > HBA > SSA > GWO > RPSO > AO > AOA > HPSBA > SCQSSA.

The empirical results in Table 3, Table 4, Table 5 and Table 6 indicate a modest increase in the optimization time of EKSSA compared to the basic SSA. This observed difference aligns with the conclusions drawn from the theoretical computational complexity analysis. For F1 with high dimension, the consumption time of the designed EKSSA is 1.6713E-01 s and the consumption time of the SSA is 1.6116E-01 s, which is approximately 0.006 s higher. For the F16 with fixed dimension, the consumption times of EKSSA and SSA are 2.7603E-01 s and 2.7019E-01 s, respectively, which is also approximately 0.006 s higher. Although the computational complexity is slightly higher, the performance of the proposed EKSSA is significantly improved compared to SSA from the results of the test functions.

**Figure 3:**
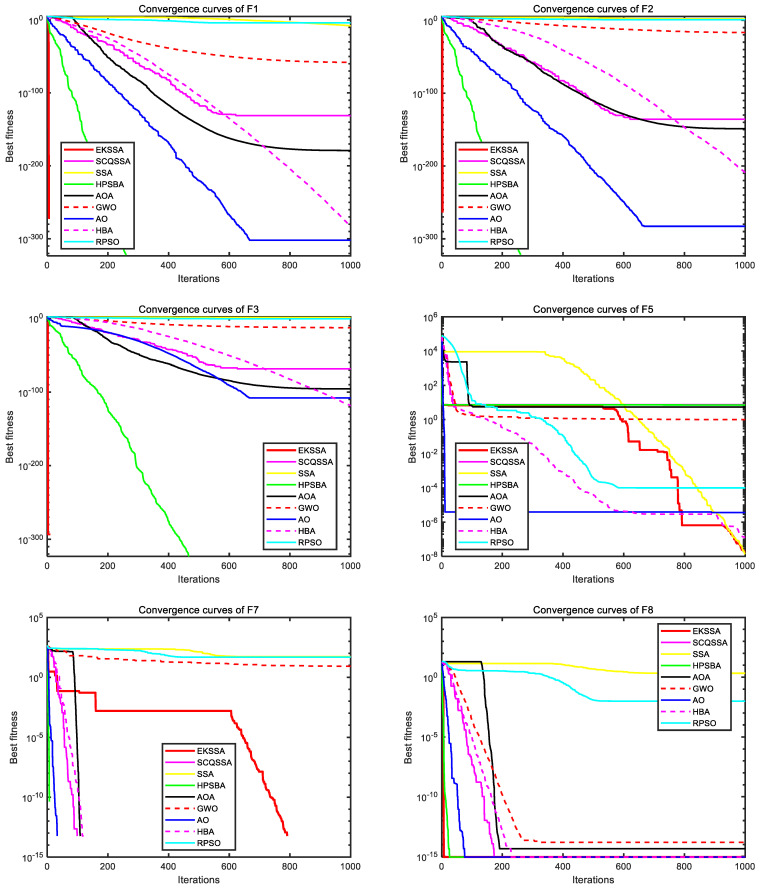
Convergence curves of the comparison algorithms on F1 to F3, F5, F7, and F8.

**Figure 4:**
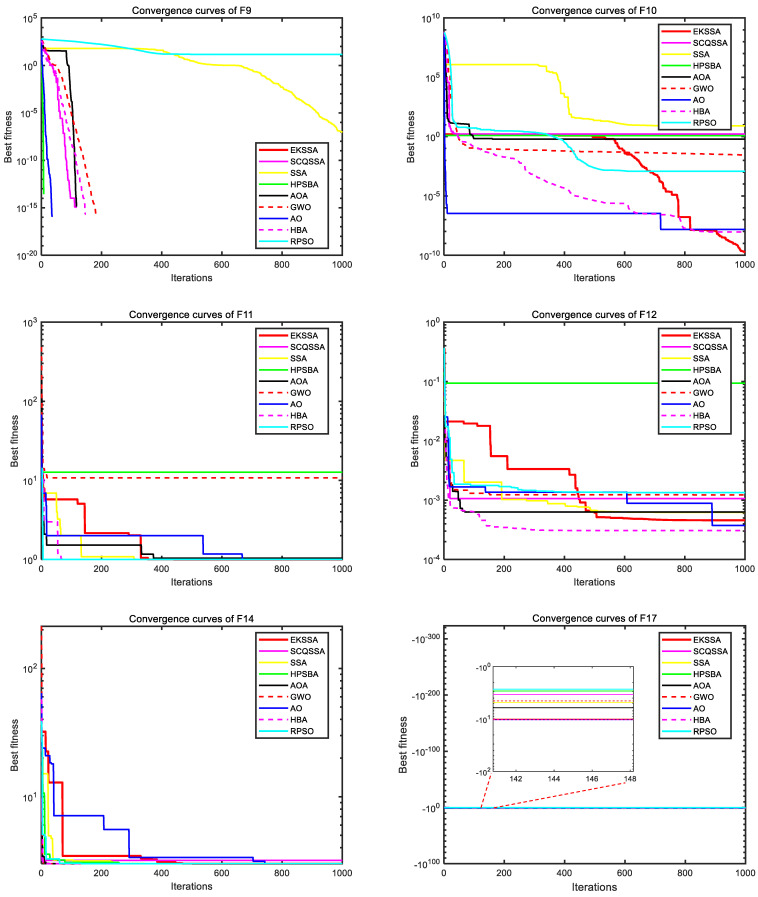
Convergence curves of the comparison algorithms on F9 to F12, F14, and F17.

Figure 3 and Figure 4 depict the convergence curves, which can be used to analyze the convergence speed and accuracy curve of comparison methods. From Figure 3, the EKSSA has better convergence than comparison methods with a fast speed on F1, F2, F3, and F8. From Figure 4, for F10, the convergence curve of EKSSA obtains the best value. For F5, F7, F10, and F12, the proposed EKSSA curves have multiple inflection points, indicating that it has a very good ability to escape from local optima. For other functions, the proposed EKSSA does not outperform all peers, indicating potential for further enhancement of its convergence properties in future work.

### 4.4. Boxplot Results Analysis

**Figure 5:**
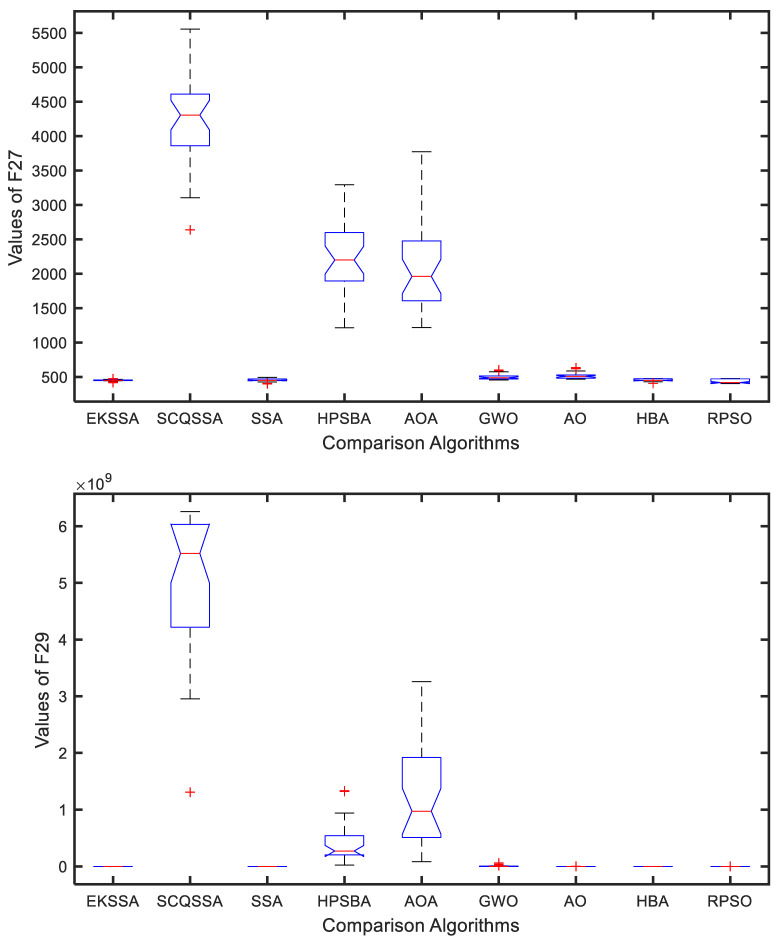
Boxplot for F27 and F29 of the comparison methods.

**Figure 6:**
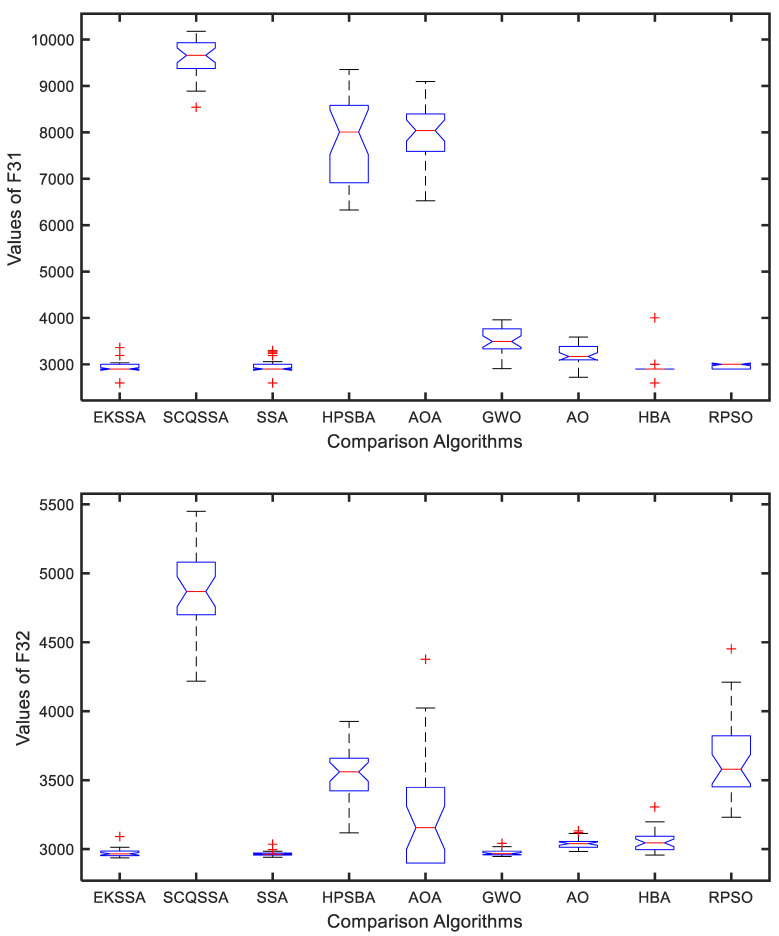
Boxplot for F31 and F32 of the comparison methods.

The boxplot can be used to explain the stability of the comparison approaches. Figure 5 and Figure 6 show the boxplot results of the nine comparison methods in F27, F29, F31, and F32. Notably, each algorithm was independently run 30 times for a test function. From Figure 5, EKSSA is better than others in F27. EKSSA, SSA, and HBA have a similar result to the boxplot of F29. In Figure 6, there are a few outliers in EKSSA from F31 and F32. The boxplot results demonstrate that the proposed EKSSA has better stability performance for the numerical optimization problem.

### 4.5. Ablation Results Analysis

To evaluate the contribution of each proposed strategy, ablation studies were conducted, and the results are summarized in Table 7. In this table, EKSSA1 denotes the algorithm that incorporates only adaptive adjustment strategies for parameters $c_{1}$ and α. EKSSA2 corresponds to the version that uses solely the novel position update strategy via Gaussian walk. EKSSA3 represents the configuration with only the dynamic mirror learning strategy. For comparison, the baseline SSA and fully integrated EKSSA are also included, which combines all three strategies. From Table 7, EKSSA1, EKSSA2, and EKSSA3 are all superior to basic SSA, indicating that the proposed improvement strategy is effective. The EKSSA that combines multiple strategies has the best performance, which indicates that the fusion of multiple strategies can effectively enhance the optimization ability of the algorithm and has a complementary effect.

## 5. Results of EKSSA-SVM for Seed Classification

High-quality seeds can help farmers achieve better profits and safe food. The classification of seeds through intelligent technology not only improves efficiency but also reduces the cost of manual screening. Thus, it is necessary for us to study the ML algorithm to identify seed varieties. The efficiency and accuracy of existing methods still hold potential for further improvement. Notably, there is also a gap for the seed classification task by the optimizing the hyperparameter of SVM using SIAs. In this research, two seed classification datasets were used to verify the performance of the proposed EKSSA. The SVM [37] served as the baseline model for seed classification, and the EKSSA was employed to optimize its hyperparameters, specifically the penalty coefficient *c* and the kernel parameter *g*.

**Figure 7:**
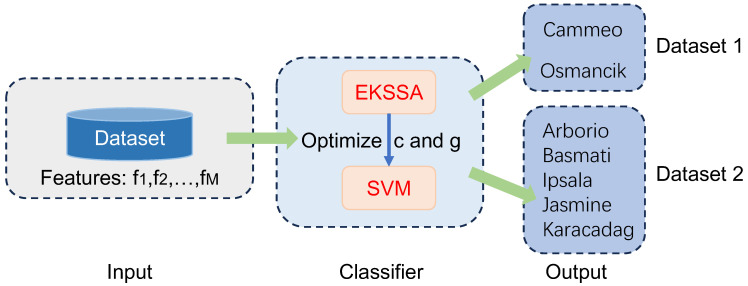
The diagram of the EKSSA-SVM for seed classification.

Figure 7 presents the diagram of EKSSA-SVM for seed classification tasks using two Rice Varieties datasets from the open resources. There are two categories in the Rice Varieties Dataset 1, 1630 Cammeo and 2180 Osmancik [38]. Seven features ($f_{1},f_{2},\ldots ,f_{M}$, M=7) were sourced and consolidated with Area, Diameter, Major-axis length, Minor-axis length, Eccentricity, Convex-area, and Extent. In addition, Rice Varieties Dataset 2 has five categories: Arborio, Basmati, Ipsala, Jasmine, and Karacadag [39]; 10,000 samples were used for each category in this study. Sixteen features ($f_{1},f_{2},\ldots ,f_{M}$, M=16) were sourced and consolidated with Area, Perimeter, Major-Axis, Minor-Axis, Eccentricity, Eqdiasq, Solidity, Convex-Area, Extent, Aspect-Ratio, Roundness, Compactness, Shapefactor1, Shapefactor2, Shapefactor3, and Shapefactor4. The metric accuracy (Acc/%) is defined as:

(13) $$Acc=\frac{TP+TN}{TP+TN+FP+FN}\times 100\%,$$

where TP indicates the true positive number of the seed sample; TN denotes the true negative number of the seed sample. FP and FN are the false positive and negative number of the seed sample, respectively.

**Figure 8:**
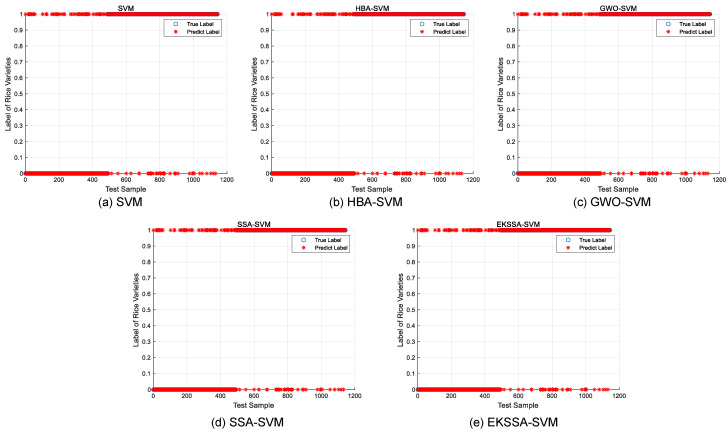
Predict results of the comparison approaches for Rice Varieties Dataset 1.

Notably, the features of the seed classification task are first normalized before being input into the classifier, which should be mapped between 0 and 1. The optimization of hyperparameters *c* and *g* significantly influences the predictive accuracy of the SVM model in the seed classification task. In these experiments, the dataset was partitioned into training and testing sets with a ratio of 7:3. The search intervals for *c* and *g* were set to [0.1, 5] and [0.1, 10], respectively. The population size was set to 10, and the iteration was set to 15. The comparison methods were selected from the top four ranking of the nine algorithms, which are EKSSA, HBA, SSA, and GWO. The results of Rice Varieties Dataset 1 and Dataset 2 are listed in Table 8 and Table 9, respectively. In addition, the true label and the predict label result is depicted in Figure 8 of the five methods.

For the Rice Varieties Dataset 1 in Table 8, test Acc (%) results of KNN, SVM, HBA-SSA, GWO-SVM, SSA-SVM, and EKSSA-SVM are 88.58, 90.5512 with c=1,g=3, 90.6387 with c=4.16158,g=1.42268, 90.8136 with c=5,g=0.187119, 90.7262 with c=4.93214,g=0.241651, and 90.8136 with c=4.99847,g=0.187177, respectively. Although the classification accuracy of EKSSA-SVM and GWO-SSA is the same, the *c* and *g* values for optimization are different.

For the Rice Varieties Dataset 2 in Table 9, the accuracy is 97.7867% by the basic SVM with c=0.3,g=2. SSA-SVM has 98.0667% Acc with c=0.331123,g=1.95375. GWO-SVM has 97.9600% Acc with c=1.43546,g=2.67443. HBA-SVM has 98.0667% Acc with c=1.27977,g=8.6252. The proposed EKSSA-SVM has 98.1133% Acc for the Rice Varieties classification task when *c* is set to 3.89383 and *g* is set to 6.03773. It is 0.3266, 0.0466, 0.1533, and 0.3466 percentage points higher than SVM, HBA-SVM, GWO-SVM, SSA-SVM, and EKSSA-SVM, respectively.

**Figure 9:**
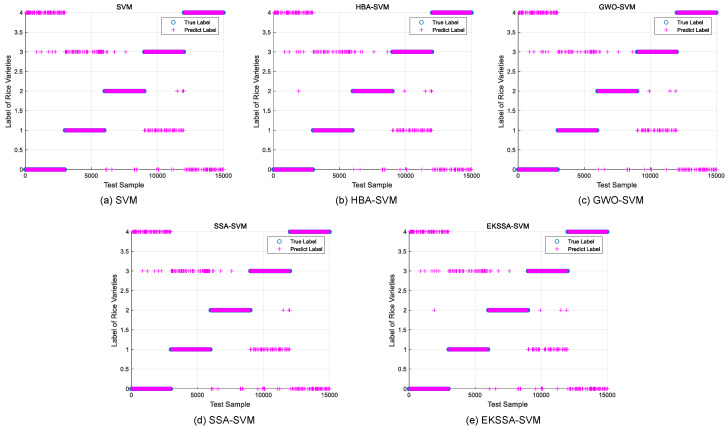
Predict results of the comparison approaches for Rice Varieties Dataset 2.

From Table 8 and Table 9, for different datasets, the values of parameter *c* and *g* are different by the proposed EKSSA-SVM. In addition, the true label and the predict label result of the five Rice Varieties dataset are depicted in Figure 9 of the SVM, HBA-SVM, GWO-SVM, SSA-SVM, and EKSSA-SVM. Except for Jasmine, there are obvious misclassification samples in the other types of Rice Varieties classification using Dataset 2. Thus, the predicted results of the EKSSA-SVM can be further modified by the quantum strategy.

## 6. Conclusions and Future Work

To address the deficiency that SSA is prone to fall into local optima, the EKSSA was designed and used to fill a gap for the seed classification task by optimizing the hyperparameter of SVM. Different adjustment strategies for the parameters $c_{1}$ and α are used to balance the exploration and exploitation of the slaps. Moreover, a novel position update strategy is inspired by the Gaussian walk theory, which improves the global search ability of the slap individual after the basic position update stage. Notably, a dynamic mirror learning strategy is designed to mirror the search range of the optimization problem with a local search capability. The performance of the EKSSA is verified by the thirty-two CEC benchmark functions, which is compared via eight advanced algorithms of RPSO, GWO, AOA, HPSBA, AO, HBA, SSA, and SCQSSA. In addition, a combined EKSSA-SVM classifier is proposed for the seed classification problem with a higher accuracy, in which EKSSA-SVM has 98.1133% Acc for the five Rice Varieties datasets when *c* is set to 3.89383 and *g* is set to 6.03773. In future work, chaotic population initialization and quantum strategies will be used to improve the performance of the EKSSA, which will be applied to solve the task of diagnosing plant leaf diseases [40].

## Figures and Tables

**Table 1:** Eighteen test functions for the performance evaluation.

Formula	Range	Dim	$f_{\min}$	Category
$F1=\sum_{i=1}^{Dim}x_{i}^{2}$	[−100, 100]	30	0	U
$F2=\sum_{i=1}^{Dim}{\left(\sum_{j=1}^{i}x_{j}\right)}^{2}$	[−100, 100]	30	0	U
$F3=\max\left\{\left|x_{i}\right|,1\leq i\leq Dim\right\}$	[−100, 100]	30	0	U
$F4=\sum_{i=1}^{Dim}\left(100{\left(x_{i+1}-x_{i}^{2}\right)}^{2}+{\left(x_{i}-1\right)}^{2}\right)$	[−30,30]	30	0	U
$F5=\sum_{i=1}^{Dim}{\left(x_{i}+0.5\right)}^{2}$	[−100, 100]	30	0	U
$F6=\sum_{i=1}^{Dim}ix_{i}^{4}+rand\left(0,\;1\right)$	[−1.28, 1.28]	30	0	U
$F7=\sum_{i=1}^{Dim}\left(x_{i}^{2}-10\cos\left(2\pi x_{i}\right)+10\right)$	[−5.12, 5.12]	30	0	M
$F8=-20\exp\left(-0.2\sqrt{\frac{1}{Dim}\sum_{i=1}^{Dim}x_{i}^{2}}\right)-\exp\left(\frac{1}{Dim}\sum_{i=1}^{Dim}\cos(2\pi x_{i})\right)+20+e$	[−32, 32]	30	0	M
$F9=\frac{1}{4000}\sum_{i=1}^{Dim}x_{i}^{2}-\prod_{i=1}^{Dim}\cos\left(\frac{x_{i}}{\sqrt{i}}\right)+1$	[−600, 600]	30	0	M
$F10=\frac{\pi }{Dim}\left\{\sum_{i=1}^{Dim-1}{\left(y_{i}-1\right)}^{2}\left[1+10\sin^{2}\left(\pi y_{i+1}\right)\right]+{\left(y_{Dim-1}\right)}^{2}+10\sin^{2}\left(\pi y_{1}\right)\right\}+\sum_{i=1}^{Dim}u(x_{i},10,100,4),y_{i}=1+\frac{x_{i}+1}{4},u_{y_{i},a,k,m}=\left\{\begin{array}{c} k{\left(x_{i}-a\right)}^{m},x_{i}>a,\\ 0,-a\leq x_{i}\leq a,\\ k{\left(-x_{i}-a\right)}^{m},x_{i}<a \end{array}\right.$	[−50, 50]	30	0	M
$F11={\left(\frac{1}{500}+\sum_{j=1}^{25}\frac{1}{j+\sum_{i=1}^{2}{\left(x_{i}-a_{ij}\right)}^{6}}\right)}^{-1}$	[−65, 65]	2	1	Fixed
$F12=\sum_{i=1}^{11}{\left[a_{i}-\frac{x_{1}\left(b_{i}^{2}+b_{i}x_{2}\right)}{b_{i}^{2}+b_{i}x_{3}+x_{4}}\right]}^{2}$	[−5, 5]	4	0.00030	Fixed
$F13=4x_{1}^{2}-2.1x_{1}^{4}+\frac{1}{3}x_{1}^{6}+x_{1}x_{2}-4x_{2}^{2}+4x_{2}^{4}$	[−5, 5]	2	−1.0316	Fixed
$F14=\left[1+{\left(x_{1}+x_{2}+1\right)}^{2}\left(19-14x_{1}+3x_{1}^{2}-14x_{2}+6x_{1}x_{2}+3x_{2}^{2}\right)\right]\times \left[30+{\left(2x_{1}-3x_{2}\right)}^{2}\times \left(18-32x_{1}+12x_{1}^{2}+48x_{2}-36x_{1}x_{2}+27x_{2}^{2}\right)\right]$	[−2, 2]	2	3	Fixed
$F15=-\sum_{i=1}^{4}c_{i}\exp\left(-\sum_{j=1}^{3}a_{ij}{\left(x_{j}-p_{ij}\right)}^{2}\right)$	[1, 3]	3	−3.86	Fixed
$F16=-\sum_{i=1}^{5}{\left[\left(X-a_{i}\right)\left(X-a_{i}\right)+c_{i}\right]}^{-1}$	[0, 10]	4	−10.1532	Fixed
$F17=-\sum_{i=1}^{7}{\left[\left(X-a_{i}\right)\left(X-a_{i}\right)+c_{i}\right]}^{-1}$	[0, 10]	4	−10.4028	Fixed
$F18=-\sum_{i=1}^{10}{\left[\left(X-a_{i}\right)\left(X-a_{i}\right)+c_{i}\right]}^{-1}$	[0, 10]	4	−10.5363	Fixed

**Table 2:** Comparison of algorithm hyperparameter settings.

Algorithms	Hyperparameter
RPSO [32]	$c_{p,max}=c_{g,max}=2.5,c_{p,min}=c_{g,min}=0.5,\omega_{min}=0.2,\omega_{max}=0.9,V_{max}=1.5$
GWO [5]	$a_{first}=2,a_{final}=0$
AOA [33]	$C_{1}=2,C_{2}=6,C_{3}=1,C_{4}=2,\mu =0.9,L=0.1$
HPSBA [34]	$a=0.1,c\left(0\right)=0.35,SP=0.6,\mu =4,\omega_{u}=0.9,\omega_{l}=0.2,C_{1}=C_{2}=2,V_{max}=1$
AO [7]	$\alpha =0.1,\delta =0.1,u=0.0265,r_{0}=10$
HBA [35]	$C=2,\beta =6,r_{1}\;to\;r_{4}\in \left(0,1\right)$
SSA [6]	$c_{2},c_{3}\in \left(0,1\right)$
SCQSSA [36]	$a=2,r=0.5,c_{2},c_{3},c_{4}\in \left(0,1\right)$
EKSSA	$\theta =0.5,c_{2},c_{3}\in \left(0,1\right),r_{1},r_{2}\in \left(0,1\right)$

**Table 3:** Comparative evaluation of nine algorithms using 18 benchmark functions.

Fun.	Item	RPSO	HBA	AO	GWO	AOA	HPSBA	SSA	SCQSSA	EKSSA
F1	Best	1.0871E-05	5.0979E-286	2.1672E-302	4.6005E-61	6.8467E-270	0.0000E+00	7.9382E-09	3.4286E-143	0.0000E+00
	Worst	4.1424E-03	3.1094E-275	7.0015E-198	4.3320E-58	2.8345E-170	0.0000E+00	2.1899E-08	5.2806E-131	0.0000E+00
	Mean	3.4513E-04	1.1435E-276	3.1090E-199	6.3414E-59	1.0401E-171	0.0000E+00	1.2965E-08	2.1250E-132	0.0000E+00
	Std	7.4244E-04	0.0000E+00	0.0000E+00	1.0112E-58	0.0000E+00	0.0000E+00	3.4796E-09	9.7067E-132	0.0000E+00
	Median	1.6531E-04	1.8544E-280	4.1845E-287	2.5257E-59	3.2191E-202	0.0000E+00	1.2252E-08	2.3128E-136	0.0000E+00
	Time/s	7.6739E-02	2.3857E-01	2.5940E-01	1.8745E-01	1.6116E-01	2.0342E-01	1.6667E-01	8.3104E-01	1.6713E-01
F2	Best	1.6665E+00	2.4658E-219	2.0469E-300	2.2524E-20	9.4293E-215	0.0000E+00	5.4015E+01	1.3441E-143	0.0000E+00
	Worst	2.5062E+01	6.5791E-202	4.6860E-193	3.3923E-14	3.1095E-130	0.0000E+00	1.2339E+03	1.8284E-125	0.0000E+00
	Mean	8.7402E+00	2.8131E-203	1.5620E-194	4.2494E-15	1.0365E-131	0.0000E+00	2.8990E+02	6.5991E-127	0.0000E+00
	Std	5.1008E+00	0.0000E+00	0.0000E+00	8.8180E-15	5.6771E-131	0.0000E+00	2.1942E+02	3.3383E-126	0.0000E+00
	Median	7.5736E+00	1.4701E-209	3.7262E-279	1.4928E-16	3.9345E-173	0.0000E+00	2.4519E+02	2.0619E-134	0.0000E+00
	Time/s/s	3.2722E-01	4.9403E-01	7.6181E-01	4.2974E-01	4.1021E-01	6.8059E-01	4.1281E-01	1.1040E+00	4.1676E-01
F3	Best	1.3808E-01	1.3574E-124	1.3873E-151	3.9650E-16	6.2441E-117	0.0000E+00	1.7994E+00	1.0777E-73	0.0000E+00
	Worst	4.4836E-01	8.7589E-118	1.2381E-101	8.8627E-14	3.2709E-79	0.0000E+00	1.6331E+01	6.5961E-65	0.0000E+00
	Mean	2.6266E-01	6.9002E-119	4.1270E-103	1.3036E-14	1.0904E-80	0.0000E+00	7.1955E+00	2.4068E-66	0.0000E+00
	Std	7.8817E-02	1.8666E-118	2.2604E-102	1.7775E-14	5.9717E-80	0.0000E+00	3.4866E+00	1.2017E-65	0.0000E+00
	Median	2.4784E-01	1.8064E-120	1.7965E-145	8.2225E-15	3.1810E-92	0.0000E+00	7.1382E+00	1.1317E-68	0.0000E+00
	Time/s	7.8774E-02	2.3851E-01	2.6371E-01	1.8261E-01	1.6240E-01	1.8497E-01	1.5898E-01	8.4002E-01	1.6551E-01
F4	Best	2.2844E+01	2.0236E+01	9.5558E-06	2.5265E+01	2.8682E+01	2.8893E+01	2.5476E+01	2.8298E+01	2.8302E+01
	Worst	9.3042E+01	2.2669E+01	3.2068E-03	2.8550E+01	2.8953E+01	2.8990E+01	1.6919E+03	2.8966E+01	2.8553E+01
	Mean	3.9698E+01	2.1840E+01	7.9130E-04	2.6775E+01	2.8868E+01	2.8945E+01	1.9731E+02	2.8842E+01	2.8492E+01
	Std	2.4343E+01	5.6739E-01	8.5900E-04	7.9266E-01	7.5559E-02	2.4464E-02	3.4706E+02	1.3987E-01	6.2818E-02
	Median	2.8633E+01	2.1824E+01	4.0502E-04	2.7038E+01	2.8910E+01	2.8951E+01	4.8841E+01	2.8860E+01	2.8507E+01
	Time/s	1.0746E-01	2.7113E-01	3.2625E-01	2.1368E-01	1.8402E-01	2.4121E-01	1.9279E-01	8.6755E-01	1.9446E-01
F5	Best	3.0734E-06	4.5395E-09	2.3206E-07	1.0059E-05	4.7257E+00	5.6910E+00	6.0874E-09	7.5000E+00	9.3936E-09
	Worst	6.4403E-03	5.4570E-07	1.1377E-04	1.5042E+00	6.1407E+00	7.2786E+00	2.0411E-08	7.5000E+00	2.2061E-08
	Mean	4.7903E-04	8.3845E-08	2.0746E-05	6.2593E-01	5.6421E+00	7.0171E+00	1.2264E-08	7.5000E+00	1.3787E-08
	Std	1.2043E-03	1.3975E-07	3.2782E-05	3.5178E-01	3.3251E-01	2.9880E-01	2.9866E-09	0.0000E+00	2.7012E-09
	Median	1.3118E-04	3.0803E-08	5.5117E-06	5.0430E-01	5.7044E+00	7.0965E+00	1.2092E-08	7.5000E+00	1.3364E-08
	Time/s	7.5309E-02	2.3935E-01	2.5858E-01	1.8246E-01	1.5366E-01	1.7462E-01	1.5705E-01	8.3671E-01	1.6495E-01
F6	Best	3.5978E-02	3.2280E-05	3.3284E-06	1.4142E-04	4.5308E-05	2.0666E-07	2.5736E-02	9.6596E-07	8.7290E-06
	Worst	1.9572E-01	6.7496E-04	4.0907E-04	2.6931E-03	7.8691E-04	1.9422E-04	1.6190E-01	1.1648E-04	8.5701E-04
	Mean	8.8336E-02	2.3684E-04	8.6043E-05	8.5170E-04	2.8454E-04	3.4069E-05	9.1888E-02	3.8346E-05	2.0673E-04
	Std	3.8992E-02	1.7220E-04	9.0615E-05	5.8252E-04	2.1355E-04	4.6980E-05	2.9178E-02	2.9220E-05	1.8859E-04
	Median	8.3680E-02	1.8803E-04	5.1356E-05	6.8982E-04	1.9379E-04	1.6572E-05	9.2231E-02	3.1997E-05	1.6195E-04
	Time/s	1.9892E-01	3.6235E-01	5.0788E-01	3.0756E-01	2.8211E-01	4.2448E-01	2.8335E-01	9.5296E-01	2.8817E-01
F7	Best	2.5183E+01	0.0000E+00	0.0000E+00	0.0000E+00	0.0000E+00	0.0000E+00	2.6864E+01	0.0000E+00	0.0000E+00
	Worst	8.8570E+01	0.0000E+00	0.0000E+00	1.1369E-13	1.3016E+02	0.0000E+00	8.7556E+01	0.0000E+00	0.0000E+00
	Mean	4.6916E+01	0.0000E+00	0.0000E+00	1.3263E-14	4.3388E+00	0.0000E+00	5.7276E+01	0.0000E+00	0.0000E+00
	Std	1.3985E+01	0.0000E+00	0.0000E+00	2.8649E-14	2.3764E+01	0.0000E+00	1.7429E+01	0.0000E+00	0.0000E+00
	Median	4.5899E+01	0.0000E+00	0.0000E+00	0.0000E+00	0.0000E+00	0.0000E+00	5.8702E+01	0.0000E+00	0.0000E+00
	Time/s	9.9330E-02	2.4508E-01	2.8035E-01	1.9433E-01	1.6327E-01	1.9794E-01	1.7681E-01	8.3219E-01	1.8004E-01
F8	Best	1.6858E-03	8.8818E-16	8.8818E-16	7.9936E-15	8.8818E-16	8.8818E-16	2.5278E-05	8.8818E-16	8.8818E-16
	Worst	2.7782E-02	8.8818E-16	8.8818E-16	2.2204E-14	1.9967E+01	8.8818E-16	4.3829E+00	8.8818E-16	8.8818E-16
	Mean	9.5237E-03	8.8818E-16	8.8818E-16	1.5810E-14	1.2645E+01	8.8818E-16	1.8865E+00	8.8818E-16	8.8818E-16
	Std	6.4119E-03	0.0000E+00	0.0000E+00	3.2854E-15	9.7857E+00	0.0000E+00	1.0589E+00	0.0000E+00	0.0000E+00
	Median	8.0054E-03	8.8818E-16	8.8818E-16	1.5099E-14	1.9963E+01	8.8818E-16	2.1201E+00	8.8818E-16	8.8818E-16
	Time/s	9.9029E-02	2.5644E-01	2.9403E-01	1.9403E-01	1.8036E-01	2.1217E-01	1.8586E-01	8.5280E-01	1.9181E-01
F9	Best	5.7578E+00	0.0000E+00	0.0000E+00	0.0000E+00	0.0000E+00	0.0000E+00	3.1355E-08	0.0000E+00	0.0000E+00
	Worst	2.0247E+01	0.0000E+00	0.0000E+00	1.5965E-02	0.0000E+00	0.0000E+00	3.6916E-02	0.0000E+00	0.0000E+00
	Mean	1.2628E+01	0.0000E+00	0.0000E+00	8.4343E-04	0.0000E+00	0.0000E+00	9.9315E-03	0.0000E+00	0.0000E+00
	Std	4.2599E+00	0.0000E+00	0.0000E+00	3.3256E-03	0.0000E+00	0.0000E+00	9.6595E-03	0.0000E+00	0.0000E+00
	Median	1.2327E+01	0.0000E+00	0.0000E+00	0.0000E+00	0.0000E+00	0.0000E+00	9.8574E-03	0.0000E+00	0.0000E+00
	Time/s	1.2764E-01	2.7863E-01	3.3153E-01	2.2109E-01	1.8755E-01	2.4667E-01	2.0475E-01	8.6659E-01	2.1296E-01
F10	Best	5.4730E-07	2.6154E-10	1.5131E-08	1.3102E-02	3.1349E-01	7.2340E-01	2.9569E-01	1.6690E+00	5.2730E-11
	Worst	3.2205E+00	2.0290E-07	4.4939E-06	7.9633E-02	1.2967E+00	1.4506E+00	1.2787E+01	1.6690E+00	2.8605E-10
	Mean	6.4483E-01	2.0073E-08	6.9802E-07	3.5659E-02	8.8141E-01	1.1766E+00	4.3187E+00	1.6690E+00	1.3092E-10
	Std	7.7438E-01	4.7279E-08	9.9589E-07	1.5691E-02	2.4309E-01	1.4058E-01	2.9416E+00	1.1292E-15	5.5764E-11
	Median	4.1377E-01	3.8722E-09	3.0096E-07	3.4152E-02	8.9735E-01	1.1993E+00	4.0085E+00	1.6690E+00	1.1875E-10
	Time/s	4.2600E-01	6.0956E-01	9.5677E-01	5.3195E-01	5.0738E-01	8.8628E-01	5.1287E-01	1.1734E+00	5.2058E-01
F11	Best	9.9800E-01	9.9800E-01	9.9800E-01	9.9800E-01	9.9800E-01	1.9921E+00	9.9800E-01	3.9933E+00	9.9800E-01
	Worst	5.9288E+00	1.0763E+01	1.0763E+01	1.2671E+01	2.3278E+00	1.2671E+01	9.9800E-01	1.2671E+01	9.9800E-01
	Mean	1.8884E+00	1.8142E+00	2.1121E+00	4.2584E+00	1.1788E+00	1.1179E+01	9.9800E-01	1.0006E+01	9.9800E-01
	Std	1.4963E+00	2.5154E+00	2.4850E+00	4.3765E+00	3.9455E-01	3.1144E+00	2.5250E-16	2.9956E+00	3.7224E-16
	Median	9.9800E-01	9.9800E-01	9.9800E-01	2.9821E+00	9.9833E-01	1.2671E+01	9.9800E-01	1.1761E+01	9.9800E-01
	Time/s	6.8657E-01	8.2461E-01	1.5141E+00	7.1329E-01	7.2139E-01	1.4306E+00	7.6984E-01	8.0659E-01	7.7094E-01
F12	Best	3.0749E-04	3.0749E-04	3.2106E-04	3.0749E-04	3.1219E-04	3.1100E-03	3.0913E-04	7.2945E-04	4.4368E-04
	Worst	1.2899E-03	2.2553E-02	6.3536E-04	2.0363E-02	1.8970E-03	1.0530E-01	2.0366E-02	1.1743E-02	2.0363E-02
	Mean	5.1152E-04	3.9645E-03	4.5475E-04	5.0545E-03	6.6017E-04	6.7976E-02	2.7495E-03	3.1134E-03	2.0420E-03
	Std	3.1782E-04	8.0783E-03	8.6007E-05	8.5930E-03	3.2773E-04	3.0996E-02	5.9782E-03	2.6091E-03	4.9868E-03
	Median	3.3371E-04	3.0749E-04	4.3970E-04	3.0756E-04	5.5994E-04	8.1765E-02	7.4679E-04	2.4190E-03	6.7069E-04
	Time/s	3.7391E-02	1.7746E-01	1.9967E-01	7.3134E-02	8.3582E-02	1.3615E-01	1.1911E-01	2.0999E-01	1.2642E-01
F13	Best	−1.0316E+00	−1.0316E+00	−1.0316E+00	−1.0316E+00	−1.0316E+00	−1.0288E+00	−1.0316E+00	−1.0285E+00	−1.0316E+00
	Worst	−1.0316E+00	−1.0316E+00	−1.0305E+00	−1.0316E+00	−1.0298E+00	−1.9676E-01	−1.0316E+00	−9.1327E-01	l−1.0316E+00
	Mean	−1.0316E+00	−1.0316E+00	−1.0315E+00	−1.0316E+00	−1.0316E+00	−8.6481E-01	−1.0316E+00	−9.9579E-01	−1.0316E+00
	Std	6.6486E-16	6.5843E-16	2.1811E-04	9.3022E-09	3.3084E-04	2.2396E-01	7.6308E-15	2.1560E-02	3.4146E-14
	Median	−1.0316E+00	−1.0316E+00	−1.0315E+00	−1.0316E+00	−1.0316E+00	−9.6487E-01	−1.0316E+00	−9.9841E-01	−1.0316E+00
	Time/s	3.3963E-02	1.6545E-01	1.8914E-01	6.2955E-02	7.7229E-02	1.3278E-01	1.1134E-01	1.6107E-01	1.2497E-01
F14	Best	3.0000E+00	3.0000E+00	3.0001E+00	3.0000E+00	3.0000E+00	3.0000E+00	3.0000E+00	3.5372E+00	3.0000E+00
	Worst	3.0000E+00	3.0000E+00	3.0538E+00	8.4000E+01	7.0164E+00	3.4475E+01	3.0000E+00	9.2105E+01	3.0000E+00
	Mean	3.0000E+00	3.0000E+00	3.0119E+00	5.7000E+00	3.4668E+00	9.4564E+00	3.0000E+00	2.3362E+01	3.0000E+00
	Std	1.6637E-15	1.8124E-15	1.2577E-02	1.4789E+01	1.1102E+00	1.1919E+01	1.0956E-13	1.8877E+01	2.2873E-13
	Median	3.0000E+00	3.0000E+00	3.0058E+00	3.0000E+00	3.0002E+00	3.0010E+00	3.0000E+00	1.9620E+01	3.0000E+00
	Time/s	2.2985E-02	1.5308E-01	1.6649E-01	5.1694E-02	6.6687E-02	1.0908E-01	1.0242E-01	1.4711E-01	1.0659E-01
F15	Best	−3.8628E+00	−3.0048E-01	−3.0048E-01	l−3.0048E-01	−3.5146E+00	−3.0048E-01	−3.0048E-01	l−3.0048E-01	−3.0048E-01
	Worst	−3.8628E+00	−3.0048E-01	−3.0048E-01	−3.0048E-01	−7.9410E-01	−3.0048E-01	−3.0048E-01	−3.0048E-01	−3.0048E-01
	Mean	−3.8628E+00	−3.0048E-01	−3.0048E-01	−3.0048E-01	−2.6868E+00	−3.0048E-01	−3.0048E-01	−3.0048E-01	−3.0048E-01
	Std	2.6962E-15	2.2584E-16	2.2584E-16	2.2584E-16	7.2118E-01	2.2584E-16	2.2584E-16	2.2584E-16	2.2584E-16
	Median	−3.8628E+00	−3.0048E-01	−3.0048E-01	−3.0048E-01	−2.7402E+00	−3.0048E-01	−3.0048E-01	−3.0048E-01	−3.0048E-01
	Time/s	4.3984E-02	1.7840E-01	2.1879E-01	8.0353E-02	7.4867E-02	1.6007E-01	1.2369E-01	1.9545E-01	1.3739E-01
F16	Best	−1.0153E+01	−1.0153E+01	−1.0153E+01	−1.0153E+01	−1.0107E+01	−4.7195E+00	−1.0153E+01	−7.8746E+00	−1.0153E+01
	Worst	−2.6305E+00	−2.6305E+00	−1.0130E+01	−5.0552E+00	−2.7971E+00	−3.0807E-01	−2.6305E+00	−1.1705E+00	−1.0153E+01
	Mean	−6.2309E+00	−9.4009E+00	−1.0149E+01	−9.1408E+00	−7.0359E+00	−1.2026E+00	−7.4807E+00	−2.6948E+00	−1.0153E+01
	Std	3.5747E+00	2.2954E+00	5.9610E-03	2.0586E+00	2.5106E+00	1.3025E+00	3.4095E+00	1.9091E+00	3.4774E-11
	Median	−5.0552E+00	−1.0153E+01	−1.0152E+01	−1.0153E+01	−7.4458E+00	−7.3822E-01	−1.0153E+01	−1.7999E+00	−1.0153E+01
	Time/s	1.8072E-01	3.2544E-01	4.9822E-01	2.1532E-01	2.2460E-01	4.3290E-01	2.7019E-01	3.5169E-01	2.7603E-01
F17	Best	−1.0403E+01	−1.0403E+01	−1.0403E+01	−1.0403E+01	−1.0399E+01	−4.8638E+00	−1.0403E+01	−5.2867E+00	−1.0403E+01
	Worst	−2.7519E+00	−2.7659E+00	−1.0374E+01	−1.0402E+01	−3.4804E+00	−3.6735E-01	−2.7519E+00	−1.0021E+00	−1.0403E+01
	Mean	−7.2807E+00	−8.7488E+00	−1.0399E+01	−1.0402E+01	−7.0277E+00	−1.9336E+00	−9.1109E+00	−2.3042E+00	−1.0403E+01
	Std	3.6770E+00	3.0586E+00	6.6812E-03	3.0980E-04	2.6085E+00	1.3987E+00	2.6831E+00	9.7513E-01	3.7219E-11
	Median	−1.0403E+01	−1.0403E+01	−1.0401E+01	−1.0402E+01	−7.5839E+00	−1.4065E+00	−1.0403E+01	−2.0145E+00	−1.0403E+01
	Time/s	2.4129E-01	3.9195E-01	6.2608E-01	2.8672E-01	2.9807E-01	5.7434E-01	3.3569E-01	4.4305E-01	3.4653E-01
F18	Best	−1.0536E+01	−1.0536E+01	−1.0536E+01	−1.0536E+01	−1.0533E+01	−4.5544E+00	−1.0536E+01	−8.1276E+00	−1.0536E+01
	Worst	−2.4217E+00	−1.8595E+00	−1.0497E+01	−1.0535E+01	−2.5691E+00	−4.8413E-01	−2.4273E+00	−9.9274E-01	−1.0536E+01
	Mean	−6.9934E+00	−8.9549E+00	−1.0532E+01	−1.0536E+01	−7.6711E+00	−2.2185E+00	−1.0086E+01	−3.0340E+00	−1.0536E+01
	Std	3.7086E+00	3.2289E+00	8.8857E-03	2.5735E-04	2.7035E+00	1.2678E+00	1.7510E+00	1.8349E+00	3.7452E-11
	Median	−7.8560E+00	−1.0536E+01	−1.0535E+01	−1.0536E+01	−8.3417E+00	−1.8073E+00	−1.0536E+01	−2.0964E+00	−1.0536E+01
	Time/s	3.2085E-01	4.7751E-01	7.7526E-01	3.5927E-01	3.6384E-01	7.0915E-01	4.0120E-01	4.9163E-01	4.1779E-01
Friedman Test		5.18	4.27	4.50	5.10	4.94	5.73	5.69	6.38	3.21
Rank		6	2	3	5	4	8	7	9	1

**Table 4:** WSR test *p*-values of eight algorithms with designed EKSSA.

Functions	RPSO	HBA	AO	GWO	AOA	HPSBA	SSA	SCQSSA
F1	1.73E-06	1.73E-06	1.73E-06	1.73E-06	1.73E-06	1.00E+00	1.73E-06	1.73E-06
F2	1.73E-06	1.73E-06	1.73E-06	1.73E-06	1.73E-06	1.00E+00	1.73E-06	1.73E-06
F3	1.73E-06	1.73E-06	1.73E-06	1.73E-06	1.73E-06	1.00E+00	1.73E-06	1.73E-06
F4	1.73E-06	1.73E-06	1.73E-06	2.13E-06	1.73E-06	1.73E-06	6.84E-03	1.92E-06
F5	1.73E-06	3.32E-04	1.73E-06	1.73E-06	1.73E-06	1.73E-06	8.59E-02	1.73E-06
F6	1.73E-06	5.86E-01	5.71E-04	1.73E-06	1.41E-01	1.97E-05	1.73E-06	1.24E-05
F7	1.73E-06	1.00E+00	1.00E+00	3.13E-02	1.00E+00	1.00E+00	1.73E-06	1.00E+00
F8	1.73E-06	1.00E+00	1.00E+00	7.99E-07	2.69E-05	1.00E+00	1.73E-06	1.00E+00
F9	1.73E-06	1.00E+00	1.00E+00	5.00E-01	1.00E+00	1.00E+00	1.73E-06	1.00E+00
F10	1.73E-06	1.73E-06	1.73E-06	1.73E-06	1.73E-06	1.73E-06	1.73E-06	1.73E-06
F11	3.68E-03	6.52E-02	1.73E-06	1.73E-06	1.73E-06	1.73E-06	1.25E-01	1.73E-06
F12	9.84E-03	2.13E-01	1.24E-05	5.58E-01	4.07E-02	1.73E-06	4.41E-01	5.29E-04
F13	1.63E-06	1.63E-06	1.73E-06	1.73E-06	1.73E-06	1.73E-06	7.00E-05	1.73E-06
F14	1.73E-06	1.73E-06	1.73E-06	1.73E-06	1.73E-06	1.73E-06	2.32E-04	1.73E-06
F15	4.32E-08	1.00E+00	1.00E+00	1.00E+00	1.73E-06	1.00E+00	1.00E+00	1.00E+00
F16	3.61E-03	2.77E-03	1.73E-06	1.73E-06	1.73E-06	1.73E-06	1.02E-01	1.73E-06
F17	1.02E-01	3.71E-01	1.73E-06	1.73E-06	1.73E-06	1.73E-06	7.97E-01	1.73E-06
F18	2.07E-02	1.65E-01	1.73E-06	1.73E-06	1.73E-06	1.73E-06	5.32E-03	1.73E-06

**Table 5:** Results of the nine comparison methods on the CEC2017 and CEC2022 benchmark functions with WSR test.

Functions	Item	EKSSA	SCQSSA	SSA	HPSBA	AOA	GWO	AO	HBA	RPSO
F19	Mean	5.00E+02	1.73E+04	5.10E+02	1.47E+04	1.19E+04	6.28E+02	6.62E+02	5.05E+02	4.74E+02
	Std	1.32E+01	2.49E+03	2.70E+01	3.92E+03	2.49E+03	1.13E+02	7.71E+01	2.79E+01	2.53E+01
	Time	2.59E-01	9.31E-01	2.53E-01	3.84E-01	2.62E-01	3.00E-01	4.77E-01	2.81E-01	1.73E-01
	*p*-value	/	1.73E-06	1.16E-01	1.73E-06	1.73E-06	1.73E-06	1.73E-06	2.71E-01	1.15E-04
F20	Mean	6.21E+02	1.00E+03	6.70E+02	9.08E+02	8.70E+02	6.27E+02	7.10E+02	6.27E+02	7.30E+02
	Std	3.77E+01	1.95E+01	3.95E+01	3.54E+01	2.52E+01	4.32E+01	3.45E+01	3.35E+01	3.38E+01
	Time	3.05E-01	9.85E-01	2.86E-01	4.44E-01	2.83E-01	3.15E-01	5.24E-01	3.08E-01	2.00E-01
	*p*-value	/	1.73E-06	6.89E-05	1.73E-06	1.73E-06	6.73E-01	1.92E-06	3.39E-01	2.35E-06
F21	Mean	6.35E+02	7.10E+02	6.50E+02	6.88E+02	6.79E+02	6.12E+02	6.48E+02	6.15E+02	6.54E+02
	Std	1.12E+01	6.30E+00	1.44E+01	7.50E+00	6.41E+00	4.94E+00	7.38E+00	6.61E+00	8.91E+00
	Time	4.01E-01	1.10E+00	4.12E-01	6.89E-01	4.05E-01	4.53E-01	7.57E-01	4.20E-01	3.19E-01
	*p*-value	/	1.73E-06	1.15E-04	1.73E-06	1.73E-06	2.13E-06	1.74E-04	1.92E-06	3.11E-05
F22	Mean	2.77E+03	4.36E+03	2.78E+03	3.61E+03	3.53E+03	2.78E+03	2.96E+03	2.81E+03	3.46E+03
	Std	3.27E+01	2.55E+02	3.06E+01	1.46E+02	1.47E+02	4.66E+01	7.72E+01	4.59E+01	1.46E+02
	Time	5.48E-01	1.22E+00	5.39E-01	9.40E-01	5.30E-01	5.59E-01	1.03E+00	5.54E-01	4.46E-01
	*p*-value	/	1.73E-06	6.14E-01	1.73E-06	1.73E-06	9.43E-01	1.73E-06	1.29E-03	1.73E-06
F23	Mean	2.93E+03	4.61E+03	2.95E+03	3.85E+03	3.87E+03	2.96E+03	3.09E+03	3.03E+03	3.43E+03
	Std	3.53E+01	2.43E+02	3.34E+01	2.17E+02	1.82E+02	5.43E+01	5.17E+01	1.51E+02	1.21E+02
	Time	5.83E-01	1.27E+00	5.81E-01	1.02E+00	5.72E-01	6.01E-01	1.11E+00	5.94E-01	4.90E-01
	*p*-value	/	1.73E-06	1.99E-01	1.73E-06	1.73E-06	1.04E-02	1.73E-06	2.41E-04	1.73E-06
F24	Mean	2.91E+03	6.06E+03	2.92E+03	5.02E+03	4.74E+03	3.00E+03	2.99E+03	2.90E+03	2.91E+03
	Std	2.96E+01	5.29E+02	2.45E+01	4.75E+02	3.92E+02	3.45E+01	2.68E+01	1.40E+01	2.13E+01
	Time	5.28E-01	1.20E+00	5.20E-01	9.09E-01	5.13E-01	5.43E-01	9.97E-01	5.42E-01	4.32E-01
	*p*-value	/	1.73E-06	8.97E-02	1.73E-06	1.73E-06	1.73E-06	1.73E-06	1.75E-02	9.43E-01
F25	Mean	3.27E+03	5.48E+03	3.26E+03	4.39E+03	3.59E+03	3.26E+03	3.39E+03	3.36E+03	3.92E+03
	Std	3.62E+01	4.81E+02	2.73E+01	4.56E+02	5.57E+02	2.58E+01	7.17E+01	1.64E+02	3.15E+02
	Time	6.99E-01	1.37E+00	6.94E-01	1.25E+00	6.83E-01	7.16E-01	1.35E+00	7.07E-01	6.04E-01
	*p*-value	/	1.73E-06	1.02E-01	1.73E-06	2.06E-01	5.17E-01	2.60E-06	2.26E-03	1.73E-06
F26	Mean	3.39E+03	7.70E+03	3.26E+03	7.01E+03	5.97E+03	3.45E+03	3.42E+03	3.24E+03	3.22E+03
	Std	6.00E+02	5.80E+02	2.07E+01	6.45E+02	1.44E+03	9.96E+01	5.61E+01	2.44E+01	2.64E+01
	Time	6.20E-01	1.30E+00	6.11E-01	1.10E+00	6.09E-01	6.39E-01	1.18E+00	6.30E-01	5.27E-01
	*p*-value	/	1.73E-06	6.56E-02	1.92E-06	3.52E-06	4.07E-05	6.32E-05	1.59E-03	1.64E-05
F27	Mean	4.52E+02	4.23E+03	4.52E+02	2.20E+03	2.05E+03	5.01E+02	5.18E+02	4.55E+02	4.41E+02
	Std	1.33E+01	5.92E+02	2.19E+01	5.18E+02	5.60E+02	3.84E+01	4.66E+01	1.69E+01	2.86E+01
	Time/s	2.11E-01	6.59E-01	2.04E-01	2.68E-01	1.70E-01	1.95E-01	3.50E-01	2.06E-01	1.08E-01
	*p*-value	/	1.73E-06	6.88E-01	1.73E-06	1.73E-06	2.60E-06	1.73E-06	2.29E-01	2.85E-02
F28	Mean	8.69E+02	1.01E+03	8.80E+02	9.68E+02	9.45E+02	8.50E+02	8.81E+02	8.53E+02	8.82E+02
	Std	2.57E+01	1.37E+01	2.61E+01	1.46E+01	1.20E+01	2.00E+01	1.66E+01	1.72E+01	2.20E+01
	Time/s	2.38E-01	6.88E-01	2.33E-01	3.26E-01	1.99E-01	2.22E-01	4.11E-01	2.33E-01	1.36E-01
	*p*-value	/	1.73E-06	4.28E-02	1.73E-06	1.73E-06	5.67E-03	3.50E-02	3.00E-02	2.07E-02
F29	Mean	1.75E+04	4.95E+09	1.00E+04	3.91E+08	1.27E+09	6.32E+06	1.64E+05	9.84E+03	4.97E+03
	Std	6.71E+03	1.27E+09	7.27E+03	3.47E+08	8.74E+08	1.32E+07	1.36E+05	9.16E+03	3.58E+03
	Time/s	2.17E-01	6.71E-01	2.15E-01	2.85E-01	1.79E-01	2.01E-01	3.67E-01	2.09E-01	1.14E-01
	*p*-value	/	1.73E-06	1.38E-03	1.73E-06	1.73E-06	1.11E-02	1.73E-06	3.61E-03	1.73E-06
F30	Mean	4.14E+03	7.65E+03	3.95E+03	5.42E+03	4.76E+03	3.42E+03	3.31E+03	4.08E+03	4.43E+03
	Std	1.27E+03	7.93E+02	1.25E+03	1.68E+03	1.56E+03	6.77E+02	1.02E+03	1.03E+03	7.83E+02
	Time/s	3.03E-01	7.51E-01	2.96E-01	4.54E-01	2.62E-01	2.85E-01	5.41E-01	2.94E-01	1.98E-01
	*p*-value	/	2.13E-06	6.73E-01	4.39E-03	6.87E-02	1.48E-02	8.73E-03	9.26E-01	3.18E-01
F31	Mean	2.93E+03	9.59E+03	2.96E+03	7.87E+03	7.96E+03	3.49E+03	3.21E+03	2.94E+03	2.95E+03
	Std	1.36E+02	3.82E+02	1.77E+02	8.99E+02	6.39E+02	2.96E+02	2.12E+02	2.21E+02	5.07E+01
	Time/s	3.98E-01	8.42E-01	3.92E-01	6.39E-01	3.53E-01	3.81E-01	7.29E-01	3.93E-01	2.91E-01
	*p*-value	/	1.73E-06	7.81E-01	1.73E-06	1.73E-06	4.73E-06	1.97E-05	7.97E-01	4.07E-02
F32	Mean	2.97E+03	4.86E+03	2.97E+03	3.55E+03	3.25E+03	2.97E+03	3.04E+03	3.06E+03	3.66E+03
	Std	3.02E+01	2.80E+02	1.84E+01	2.08E+02	4.20E+02	2.18E+01	3.73E+01	7.94E+01	2.85E+02
	Time/s	4.29E-01	8.69E-01	4.20E-01	6.96E-01	3.80E-01	4.07E-01	7.90E-01	4.18E-01	3.16E-01
	*p*-value	/	1.73E-06	4.91E-01	1.73E-06	9.84E-03	4.41E-01	6.98E-06	2.60E-05	1.73E-06

**Table 6:** Friedman test results of the nine comparison methods on the CEC2017 and CEC2022 benchmark functions.

Functions	EKSSA	SCQSSA	SSA	HPSBA	AOA	GWO	AO	HBA	RPSO
F19	2.63	8.60	3.23	8.10	7.30	5.13	5.73	2.80	**1.47**
F20	**2.10**	8.97	3.70	7.77	7.27	2.40	4.90	2.53	5.37
F21	3.40	9.00	4.80	7.87	7.07	**1.37**	4.63	1.70	5.17
F22	2.27	9.00	2.40	7.43	7.00	**2.17**	5.00	3.17	6.57
F23	**2.00**	9.00	2.27	7.50	7.47	2.77	4.70	3.30	6.00
F24	2.47	8.90	3.20	7.73	7.37	5.47	5.53	**1.87**	2.47
F25	3.07	8.90	**2.53**	7.70	3.60	2.97	5.07	4.20	6.97
F26	3.43	8.70	3.03	7.90	6.90	5.70	5.47	2.30	**1.57**
F27	2.53	8.97	2.90	7.63	7.40	4.97	5.47	2.80	**2.33**
F28	3.17	9.00	4.40	7.87	7.00	**2.03**	4.40	2.43	4.70
F29	3.83	8.97	2.83	7.13	7.90	4.40	5.60	2.57	**1.77**
F30	4.17	8.87	4.17	6.43	5.57	**3.10**	**3.10**	4.43	5.17
F31	**2.37**	8.97	2.63	7.60	7.43	5.60	4.67	2.70	3.03
F32	2.57	9.00	**2.53**	6.97	4.23	2.70	4.83	4.77	7.40
Friedman Test	**40.00**	124.83	44.63	105.63	93.50	50.77	69.10	41.57	59.97
Mean Value	**2.86**	8.92	3.19	7.55	6.68	3.63	4.94	2.97	4.28
Rank	1	9	3	8	7	4	6	2	5

**Table 7:** Ablation results of the proposed strategies of the EKSSA.

Function	Item	SSA	EKSSA1	EKSSA2	EKSSA3	EKSSA
F3	Best	1.80E+00	1.52E+00	1.68E-34	5.72E-08	0.00E+00
	Worst	1.63E+01	2.07E+01	6.56E-06	1.45E-07	**0.00E+00**
	Mean	7.20E+00	7.70E+00	3.70E-06	9.48E-08	**0.00E+00**
	Std	3.49E+00	4.26E+00	1.71E-06	2.19E-08	**0.00E+00**
	Median	7.14E+00	6.56E+00	3.47E-06	9.27E-08	**0.00E+00**
	Time/s	1.59E-01	1.58E-01	1.53E-01	1.61E-01	1.66E-01
F8	Best	2.53E-05	2.47E-05	2.29E-09	**4.04E-08**	8.88E-16
	Worst	4.38E+00	4.30E+00	8.58E-06	8.18E-08	**8.88E-16**
	Mean	1.89E+00	2.15E+00	2.43E-06	5.65E-08	**8.88E-16**
	Std	1.06E+00	1.02E+00	1.73E-06	9.46E-09	**0.00E+00**
	Median	2.12E+00	2.41E+00	2.13E-06	5.57E-08	**8.88E-16**
	Time/s	1.86E-01	1.84E-01	1.80E-01	1.80E-01	1.92E-01

**Table 8:** Comparison results of the two Rice Varieties datasets.

Algorithms	Train Acc (%)	Test Acc (%)
KNN [38]	-	88.5800
SVM	-	90.5512
HBA-SSA	96.9383	90.6387
GWO-SVM	95.2958	**90.8136**
SSA-SVM	95.3139	90.7262
EKSSA-SVM	95.2957	**90.8136**

**Table 9:** Comparison results of the five Rice Varieties datasets.

Methods	Train Acc (%)	Test Acc (%)
SVM	-	97.7867
HBA-SVM	99.9999	98.0667
GWO-SVM	99.9711	97.9600
SSA-SVM	99.8947	97.7667
EKSSA-SVM	99.9999	**98.1133**

## Data Availability

The Rice Varieties datasets can be found at https://www.muratkoklu.com/datasets/.
